# Hybridization of *Oxalis corniculata* and *O. dillenii* in their non-native range

**DOI:** 10.3897/phytokeys.178.61031

**Published:** 2021-05-18

**Authors:** Quentin Groom, Sofie Meeus, Steven B. Janssens, Leen Leus, Ivan Hoste

**Affiliations:** 1 Meise Botanic Garden, Nieuwelaan 38, 1860, Meise, Belgium Meise Botanic Garden Meise Belgium; 2 Biology Department, KU Leuven, Belgium KU Leuven Leuven Belgium; 3 ILVO, Plant Sciences Unit, Flanders Research Institute for Agriculture, Fisheries and Food, Caritasstraat 39, 9090, Melle, Belgium Flanders Research Institute for Agriculture, Fisheries and Food Melle Belgium

**Keywords:** DNA content, *Oxalis* × *vanaelstii*, pollen, stomata

## Abstract

Many species have been introduced beyond their native ranges and many have become global weeds. Human mediated dispersal has removed the geographic isolation of these species, reversing millions of years of independent evolution. Examples are the Oxalis species in section Corniculatae where several species have become invasive. Here we characterize and formally describe a hybrid between *O. dillenii* and *O. corniculata*, which occurs spontaneously in Belgium and Japan. *Oxalis corniculata* is probably native to Japan, but both species are alien to Belgium and *O. dillenii* is native to North America. We formally name this hybrid as Oxalis × vanaelstii. Although this hybrid is sterile, it is nevertheless vigorous and perennial. Both parent species grow as weeds in gardens; therefore, it is likely to be more common than currently appreciated in countries where these species co-occur.

## Introduction

The human mediated redistribution of plant species, whether by accident or design, facilitates the hybridization of species that were once separated by millennia of independent evolution (Largiadèr 2008). Hybridization is a major force in the evolution of plants and the fingerprint of this breakdown in geographic isolation is likely to be marked on the future evolution of plants (Vallejo-Marín and Hiscock 2016). Hybrids can occur in a geographic range where one parent is native and the other is alien (e.g. Ayres and Strong 2001), but also between two species brought together in a range where they are both alien (e.g. Vallejo-Marín 2012).

The genus *Oxalis* is one of the weediest genera and many species have become naturalized beyond their native ranges (Pyšek et al. 2017). Therefore, *Oxalis* species from all over the world are being brought together with sibling species where they may have the opportunity to cross pollinate.

Molecular genetic techniques have shown that hybridization has been an important force in the evolution of *Oxalis* (Emshwiller and Doyle 2002; Vaio et al. 2013; Vaio et al. 2016). However, few hybrids from the genus have been formally described and, as Salter (1944) and du Preez et al. (2018) point out, field observations of hybrids in the genus are rare.

Studies have indicated that hybridization can occur between species in the section Corniculatae (Mulcahy 1965; Doust et al. 1981). For example, artificial crosses between *O. corniculata* L. and *O. dillenii* Jacq. were reported to be fertile irrespective of the direction of the crossing (Mulcahy 1965). However, only recently have sterile wild hybrids of *O. dillenii* and *O. corniculata* been characterized from Japan (Fukatsu et al. 2018). *Oxalis dillenii* is a North American species, which has naturalized in other parts of the world, including Japan and Europe (Mito and Uesugi 2004; Groom et al. 2017). *Oxalis corniculata* is now a cosmopolitan species, probably native to Japan (Groom et al. 2019).

Fukatsu et al. (2018) characterized hybrid plants from four sites in mixed populations of *O. corniculata* and *O. dillenii*. These plants are sterile and have intermediate characters of their stipules, stem hairs and floral morphology. They also have an intermediate chromosome count and DNA content. In the summer of 2016 a putative hybrid plant was found in a mixed population of *O. dillenii* and O. corniculata var. atropurpurea Planch. from Belgium. Here we characterize that plant and formally describe the hybrid. Following article H.3.1 of the International Code of Nomenclature for algae, fungi, and plants (Shenzhen Code) we propose to formally name this hybrid so that it can be unambiguously referred to (Turland et al. 2018).

## Material and methods

### Hybrid origin and habitat

For some years, one of the authors (IH) has maintained a small living collection of *Oxalis* species in his home garden in Aalter (Belgium, prov. of East Flanders). Among these plants are Oxalis corniculata var. atropurpurea and *O. dillenii*, both collected as weeds in plant containers offered for sale in garden centers and nurseries (although the former taxon was already present in the garden as a weed years before additional plants were deliberately introduced for cultivation). The precise origin of the cultivated plants is not known. Over the years, plants have escaped from the pots in which they are cultivated, as ripe seeds are catapulted away over distances that frequently exceed 1 m. It is among these escapees that, in 2016, putative hybrids were detected (voucher BR0000025668254V, see the taxonomic description). A few more plants of the possible hybrid were also found in 2017 and 2018. They grew next to plants of the two parents and flowered abundantly, yet produced no fruits. They were observed in a neglected part of the garden where selective manual weeding created opportunities for several different *Oxalis* to maintain themselves among competitors such as *Epilobium* sp., *Poa annua* and other common weeds. The hybrid grew on moderately damp, sandy, humus-rich soil. We note that all populations are homostylous.

### Additional material examined

*Oxalis dillenii*

Belgium. • Museumstraat 93, Bellem, Aalter; 51°05'44.46"N, 3°29'47.21"E; 8 Aug. 2013; Ivan Hoste 13023 (BR (BR0000013236892)).

Oxalis corniculata var. atropurpurea

Belgium. • Museumstraat 93, Bellem, Aalter; 51°05'44.46"N, 3°29'47.21"E; 2 Jul. 2019; Ivan Hoste (BR (BR0000025959222V)).

### Molecular protocols and sequence analysis

We applied a modified CTAB protocol for total genomic DNA isolation (Tel-Zur et al. 1999). Secondary metabolites were removed by washing ground, silica-dried leaf material with 1000 µL extraction buffer (100 mM Tris-HCl pH 8, 5 mM EDTA pH 8, 0.35 M sorbitol). Samples were incubated at 60 °C (1 h) with a CTAB lysis buffer (incl. 1% PVP-40 and 0.3% 2-mercaptoethanol). Extraction was done twice with SEVAG (chloroform-isoamylalcohol (24/1 v/v)) and was followed by an isopropanol precipitation (0.8 volumes). After centrifugation, the pellet was washed in 70% ethanol, air-dried, and dissolved in 50 µL buffer (10 mM Tris-HCl pH 8, 1 mM EDTA pH 8). Amplification and sequencing of ITS and *trnL-F* was performed using the primers of White et al. (1990) and Taberlet et al. (1991), respectively. PCR reactions of ITS and *trnL-F* were carried out using a touchdown PCR protocol (25 µL). PCR reactions were initiated with a 3-min heating at 95 °C followed by 20 cycles consisting of a denaturation step of 30 s at 95 °C, a 30 s annealing step starting at 58 °C decreasing each cycle with 0.5 °C, and an extension step of 72 °C for 60 s, and ending with 10 cycles (similar in timing as with the first 20 cycles) at an annealing temperature of 48 °C. PCR products were enzymatically purified using the ExoSap protocol and sequenced by the Macrogen sequencing facilities (Macrogen Europe, Amsterdam, Netherlands). Contiguous sequences were assembled using Geneious v7.0.6 (Biomatters, New Zealand). Automatic alignments were carried out with MAFFT (Katoh et al. 2002) under an E-INS-i algorithm, a 100 PAM/k = 2 scoring matrix, a gap open penalty of 1.3 and an offset value of 0.123. Subsequent manual fine-tuning of the aligned dataset was done in Geneious v7.0.6. Gaps were treated as missing data. All sequences and sample information were uploaded to European Nucleotide Archive and can be found under the project number PRJEB41412.

### Flow cytometry

Genome sizes were measured with a Partec PAS III flow cytometer equipped with an 20 mW 488 nm solid state laser. Samples were prepared using the commercial kit Cystain PI absolute P (Partec, Germany). Each of the three plants of interest, *O. corniculata*, *O. dillenii* and the new hybrid were analyzed separately and chopped with a sharp razor blade at room temperature together with an internal standard (*Zea mays*, 2C = 5.43 pg, Lysák and Doležel (1998)). The nuclei suspension was filtered through a 50 μm mesh CellTrics disposable filter (Partec GmbH, Münster, Germany), stained with propidium iodide following the specifications of the kit. At least five replicates per sample were incubated for at least 30 min in the dark before measuring with the flow cytometer on two (*O. corniculata*) to three (*O. dillenii*, hybrid) different days using the FloMax software (Quantum Analysis, Germany). Average genome size was calculated from the relative fluorescence intensities of the sample of interest and the internal standard with known genome size.

### Guard cell length

Epidermal leaf impressions were made from the abaxial side of the leaves in the middle of the leaf, between the midvein and edge. Transparent nail polish (Bourjois Crystal ball) was used to make the impressions which, once dried, were mounted pointing upward with double-sided tape (Scotch) on a microscope slide.

Stacked photomicrographs were taken per leaf print (view fields = 0.09 mm^2^) using a digital microscope (VH-5000 Ver 1.5.1.1, Keyence Corporation) with full coaxial lighting and default factory settings for shutter speed at ×1000 lens magnification (VH-Z250R).

### Pollen electron microscopy and viability count

Anthers were collected from mature buds and no distinction was made between the two whorls of anthers. Material for scanning electron microscopy was washed in 70% ethanol for 20 minutes and washed twice with 100% DMM (dimethoxymethane) for 20 minutes while being sonicated each time for a couple of seconds. Then it was washed with 100% acetone. The material was critical point dried using liquid CO_2_ with a Leica EMCPD3000 critical point dryer. The dried samples were mounted on aluminum stubs using carbon adhesive tape and coated with a platinum palladium mix with a Cressington JFC-2300/208HR sputter coater. SEM images were obtained with a JEOL JSM7100F field emission scanning electron microscope. Pollen size was measured on 200 grains for each parent and for the hybrid. The diameter of the roughly spherical pollen was measured horizontally on the photograph regardless of orientation of the pollen grain. Pollen viability was measured using the staining protocol of Peterson et al. (2010). Viability was evaluated by counting the proportion of stained versus unstained pollen grains. More than 100 grains were counted per flower for a total of three flowers.

## Results

### Morphology

**Figure 1.**
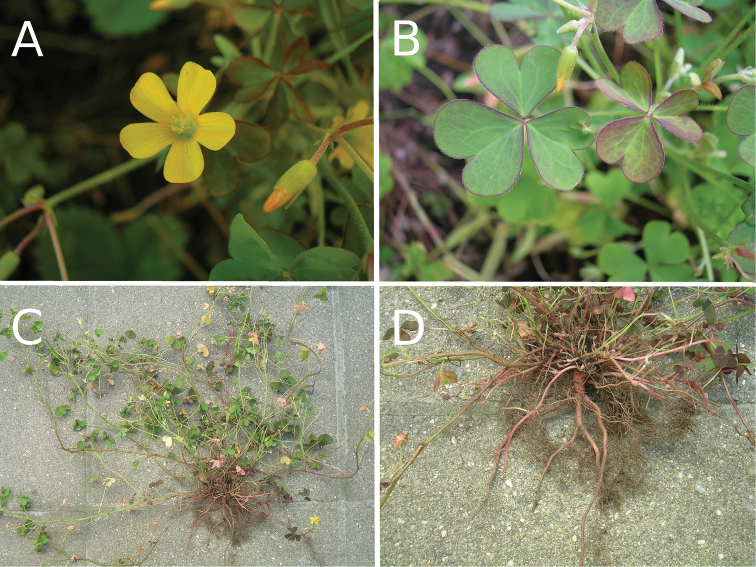
Photographs of the putative hybrid **A** flower and bud **B** leaves showing their intermediate coloration between the green *O. dillenii* and purple O. corniculata var. atropurpurea**C** whole plant showing its straggly habit **D** root system and stoloniferous shoots.

**Figure 2.**
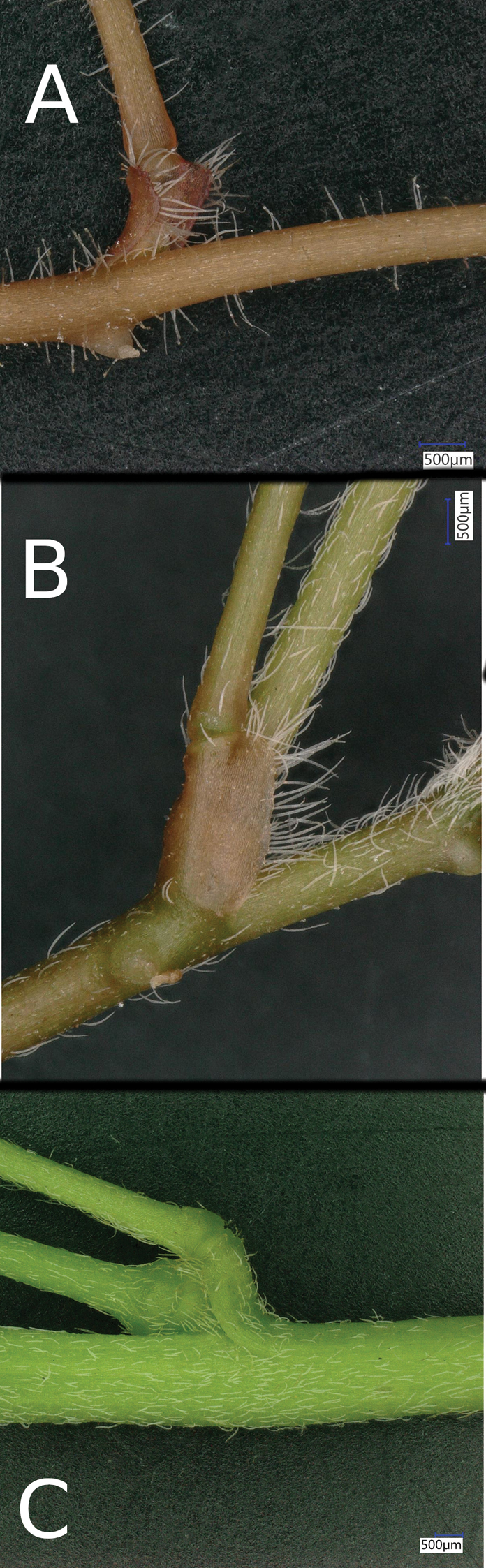
The leaf nodes of (**a**) *Oxalis corniculata* (**b**) O. × vanaelstii and (**c**) *O. dillenii* showing the stipules and stem hairs and for the latter two the peduncle.

Oxalis corniculata var. atropurpurea has purple-brown leaves and *O. dillenii* bright green leaves (Figs 1, 2). The hybrid is intermediate with pale purple-brown leaves and sometimes green leaves with a purple underside. Guard cells of *O. corniculata* are about 30% larger than those of *O. dillenii* (Table 1). However, the guard cells of the hybrid are almost exactly the same size as those of *O. dillenii*. The hybrid also has an intermediate growth form. *Oxalis dillenii* is generally erect, though it becomes decumbent with age because the main stem tends to topple over once the crown becomes too heavy. Oxalis corniculata var. atropurpurea is largely prostrate and roots at the nodes. The hybrid also roots at the nodes, but is not as strongly creeping as Oxalis corniculata var. atropurpurea (Fig. 1).

**Table 1.** Guard cell length (µm), pollen diameter (µm) and total (2C) DNA content (pg) for *O. corniculata*, O. × vanaelstii and *O. dillenii*.

	*O. corniculata*	O. × vanaelstii	*O. dillenii*
Guard cell length (µm)	23.9 (2.0, n = 45)	18.0 (1.9, n = 119)	18.4 (2.2, n = 39)
Pollen diameter (µm)	31.0 (3.1, n = 200)	20.2 (6.2, n = 200)	26.5 (2.6, n = 200)
Total DNA content 2C (pg)	2.13 (0.03, n = 5)	1.50 (0.06, n = 8)	0.88 (0.05, n = 7)

The stipules and stem of the putative hybrid are illustrated in Figure 2. *Oxalis corniculata* has short adnate stipules with an angular tip. *Oxalis dillenii* has narrow stipules with a more rounded tip. The hybrid is intermediate, though perhaps closer to *O. corniculata*. The stipule is distinct, longer than wide, but with more of a rounded tip than *O. corniculata*.

Stem and petiole hairs are also visible on Figure 2. In Oxalis corniculata var. atropurpurea they are patent and sometimes glandular, whereas in *O. dillenii* they are antrorse, eglandular and appressed to the stem or petiole. The hybrid is again intermediate in character with eglandular, antrorsely directed hairs, but they are not as strongly appressed as in *O. dillenii*.

### Cellular DNA content

Total DNA content (2C) is 0.88 pg (SD = 0.05, n = 7) for the *O. dillenii* parent, 2.13 pg (SD = 0.03, n = 5) for the *O. corniculata* parent and an intermediate DNA content of 1.50 pg (SD = 0.06, n = 8) for the putative hybrid.

### Pollen

**Figure 3.**
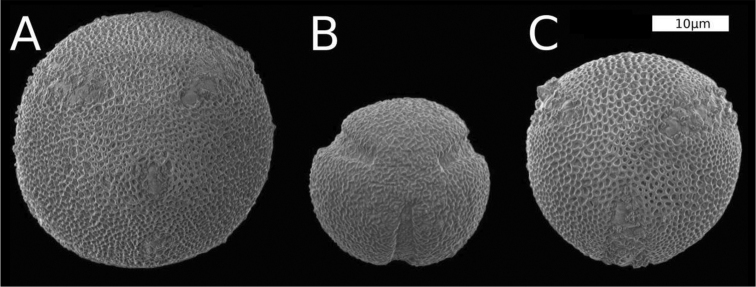
Scanning electron micrographs of pollen adjusted to the same scale **A***Oxalis corniculata***B**O. × vanaelstii**C***O. dillenii*.

Representative pollen grains of the parents and the hybrid are illustrated in Figure 3. All taxa have significantly different pollen diameters (P < 0.01) based upon a pairwise comparison using Wilcoxon rank sum test. The diameter of *O. corniculata* pollen is larger (mean 31 µm, SD = 3.1 µm, n = 200) than *O. dillenii* pollen (mean 26 µm, SD = 2.6 µm, n = 200) (Fig. 3). On average, both parents have larger pollen grains than the putative hybrid (mean 20 µm, SD = 6.2 µm, n = 200). However, note the large standard deviation and broad spread of hybrid pollen in Figure 3. This is both because some grains are exceedingly small and because about 4% of hybrid pollen grains exceed 40 µm. In pollen viability staining pollen of the parents are strongly stained and all appear to be viable. Viability staining of the pollen of the putative hybrid is 45.6% (SD = 3.0%, n = 3 flowers). Those pollen of the hybrid that were exceptionally large were seen to be stained strongly whereas small grains remained unstained.

**Figure 4.**
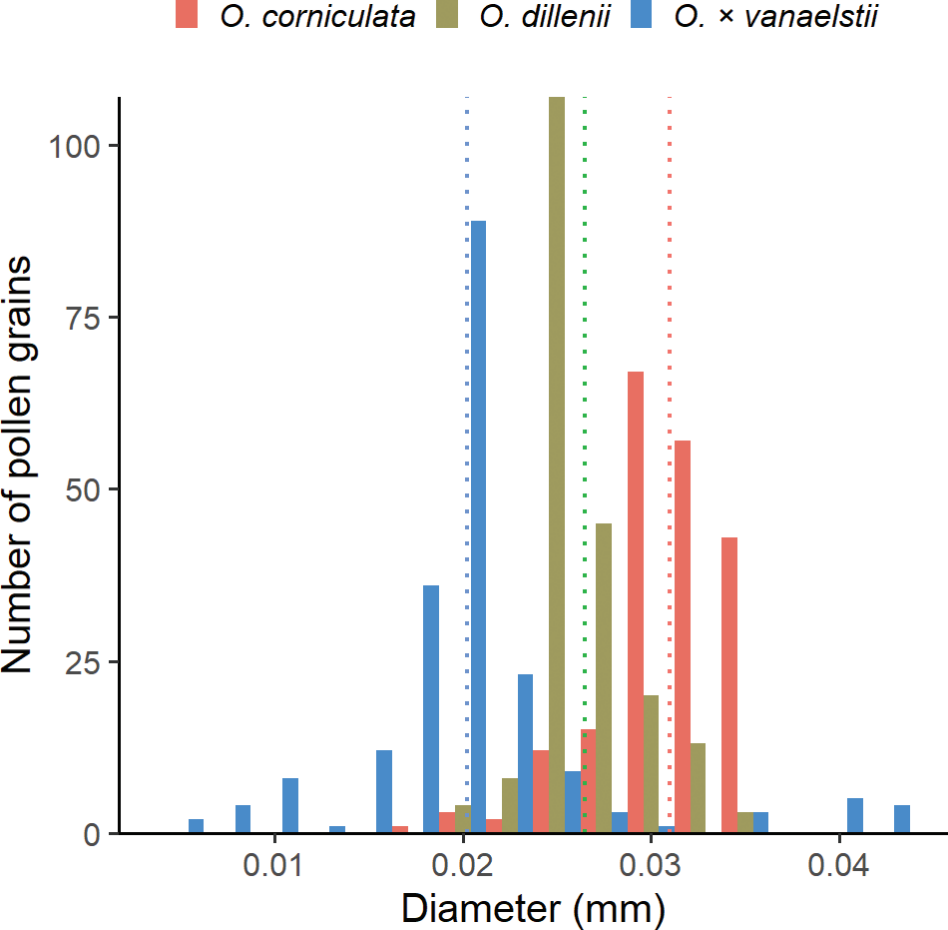
A histogram of pollen size measurements made from scanning micrographs. The dotted line indicates the mean diameter of the pollen grains.

### Sequence analysis results

**Figure 5.**
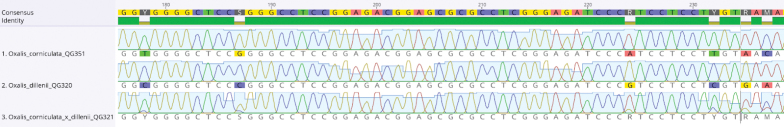
Part of the aligned electropherogram of the nuclear ribosomal marker ITS for the hybrid taxon O. corniculata × dillenii and its putative parental accessions *Oxalis corniculata* (QG351) and *O. dillenii* (QG320). Double peaks and nucleotide ambiguity codes (R,Y,M,S) indicate that taxon QG321 has been the result of hybridization. Numbers above the sequences refer to nucleotide positions.

ITS polymorphisms between *O. corniculata* and *O. dillenii* occurred at nucleotide positions 51, 52, 102, 103, 115, 122, 130, 138, 152, 165, 178, 187, 224, 232, 235, 237, 379, 423 (Fig. 5). ITS sequencing of the putative hybrid shows a clear ambiguity at each of the aforementioned polymorphic loci consistent with both parental alleles (Fig. 5). We also note a frameshift at position 423 where there are four cytosine base pairs in *O. dillenii*, versus five cytosine base pairs in *O. corniculata*. This frameshift blurs the sequencing read in the hybrid beyond this point. The chloroplastic marker *trnL-F* has a single polymorphism at position 279 (Suppl. material 1: Fig. S1). At this position, *Oxalis corniculata* has a guanine as character state whereas both *O. dillenii* and the putative hybrid are characterized by a cytosine.

## Discussion

Fukatsu et al. (2018) characterized a hybrid between *Oxalis dillenii* and O. corniculata var. corniculata. It has intermediate morphological characters as does the one we describe here. Their specimens are of intermediate habit and the stipules are winged as are ours. Similarly, they found that the amount of nuclear DNA of *O. corniculata* was about twice that of *O. dillenii* with the hybrid being intermediate. Our result of 2.13 pg is in line with the genome size of between 2.11 and 2.20 pg in the octaploid *O. corniculata* as analysed by Kubešová et al. (2010) and Vaio et al. (2013). For tetraploid *O. dillenii*Kubešová et al. (2010) found a genome size of 1.01 pg, also comparable to the 0.88 pg measured in our study.

Fukatsu et al. (2018) also counted chromosomes numbers and concluded that their parental *O. corniculata* is an octoploid (2n = 48), their *O. dillenii* is a tetraploid (2n = 24) and the hybrid is a hexaploid. This is also consistent with our hybrid and parental plants. The larger size of the *O. corniculata* pollen is consistent with a larger genome. The small size of the majority of hybrid pollen is consistent with its sterility as is the poor pollen viability. Similarly, the larger guard cell size agrees with a larger genome size for *O. corniculata*. Guard cell size is generally positively correlated with genome size in vascular plants and *Oxalis* (Beaulieu et al. 2008; Jooste 2015).

The DNA sequencing results are consistent with our sterile plant being a hybrid of *O. corniculata* and *O. dillenii*. The nuclear ITS sequence has ambiguity at each of the polymorphic loci between the proposed parent species, which suggests the hybrid genome contains alleles of both parents. The chloroplastic marker, *trnL-F*, differs in only one nucleotide in *O. corniculata* and *O. dillenii*. This single polymorphism indicates that *O. dillenii* is the likely maternal parent. This also accords with the results of Fukatsu et al. (2018) who found that *O. dillenii* was the maternal parent in 85% of Japanese specimens.

Our results agree with, and add to those of, Fukatsu et al. (2018), giving further support to the parentage of these sterile putative hybrids. The *Corniculatae* are a group of similar-looking taxa with few distinctive features. The plants described here are distinctive in their sterility, however, fertile hybrids would be considerably harder to recognize, particularly as they are likely to be more closely related and perhaps more similar morphologically. In North America and Japan, where *O. dillenii* and *O. corniculata* are respectively native, there is likely to be more genetic variation and perhaps greater chance of fertile hybrids being produced. Much more work is needed to understand the extent of hybridization in this group.

## Taxonomic description

### Oxalis × vanaelstii

Taxon classificationPlantaeOxalidalesOxalidaceae

Hoste, Meeus & Groom sp. nov.

3D605985-5F75-5A24-B662-2DE1D140E5C5

urn:lsid:ipni.org:names:77217160-1

#### Type material.

***Holotype*.** Belgium. • Bellem, Aalter; 51.09°N, 3.49°E; 31 Oct. 2016; Ivan Hoste 16054 (holotype: BR (BR0000025668254V); isotype: K, isotype: MO).

***Paratypes*.** Belgium. • Cultivated at Meise Botanic Garden; Quentin Groom 19001 (BR (BR0000025668247V)). • Cultivated at Meise Botanic Garden; Ivan Hoste & Quentin Groom S.N. (BR (BR0000025668209V)).

#### Diagnosis.

Intermediate in characters between its parents *O. corniculata* and *O. dillenii*. Prostrate to ascending, mid-sized stipules, stem hairs antrorse, not tightly appressed to the stem. Flowers with at most weak orange marks in the throat, marks sometimes absent.

#### Description.

A short-lived perennial, prostrate to ascending, herb with a thin taproot. Leaves trifoliolate with three similarly sized, heart-shaped leaflets. A narrow stipule is fused to the base of the petiole and is intermediate in width between *O. corniculata* and *O. dillenii*. The stem hair density is moderate with simple, arcuate, antrorse, pointed hairs. Not strongly appressed to the stem as in *O. dillenii*. The leaves are green or purple-brown, though if purple, not as darkly colored as Oxalis corniculata var. atropurpurea. The flowers are yellow, sometimes with weak orange streaks in the throat. Fruits are unknown.

#### Etymology.

Oxalis × vanaelstii is named to commemorate the Belgian naturalist, conservationist and mycologist Etienne Vanaelst (1948–2017) who, as a volunteer collaborating with mycologists at Ghent University, contributed to a better understanding of the diversity of mushrooms, especially those growing in and around his hometown, Knesselare (prov. of East Flanders).

#### Habitat.

Gardens.

#### Distribution area.

Europe and Japan where parental distribution overlaps. The hybrid is also highly likely to occur in North America where the parents also co-occur (Eiten 1963).

## Supplementary Material

XML Treatment for Oxalis × vanaelstii

## Supplementary materials

Supplementary material 1 Figure S1 Data type image Explanation note Part of the aligned electropherogram of the plastid gene marker trnL-F for the hybrid taxon O. corniculata × dillenii and its putative parental accessions *Oxalis corniculata* (QG351) and *O. dillenii* (QG320). https://binary.pensoft.net/file/543748This dataset is made available under the Open Database License (http://opendatacommons.org/licenses/odbl/1.0/). The Open Database License (ODbL) is a license agreement intended to allow users to freely share, modify, and use this Dataset while maintaining this same freedom for others, provided that the original source and author(s) are credited.Quentin Groom, Sofie Meeus, Steven B. Janssens

Supplementary material 2 Taxonomic details of Oxalis × vanaelstii and its parents Data type COL Explanation note A Darwin Core file containing the details of Oxalis × vanaelstii, *O. dillenii* and *O. corniculata*. https://binary.pensoft.net/file/543749This dataset is made available under the Open Database License (http://opendatacommons.org/licenses/odbl/1.0/). The Open Database License (ODbL) is a license agreement intended to allow users to freely share, modify, and use this Dataset while maintaining this same freedom for others, provided that the original source and author(s) are credited.Author: Quentin Groom
